# MicroRNA-22 negatively regulates poly(I:C)-triggered type I interferon and inflammatory cytokine production via targeting mitochondrial antiviral signaling protein (MAVS)

**DOI:** 10.18632/oncotarget.12395

**Published:** 2016-10-01

**Authors:** Shengfeng Wan, Usama Ashraf, Jing Ye, Xiaodong Duan, Ali Zohaib, Wentao Wang, Zheng Chen, Bibo Zhu, Yunchuan Li, Huanchun Chen, Shengbo Cao

**Affiliations:** ^1^ State Key Laboratory of Agricultural Microbiology, Huazhong Agricultural University, Wuhan, Hubei, 430070, P. R. China; ^2^ Laboratory of Animal Virology, College of Veterinary Medicine, Huazhong Agricultural University, Wuhan, Hubei, 430070, P. R. China; ^3^ The Cooperative Innovation Center for Sustainable Pig Production, Huazhong Agricultural University, Wuhan, Hubei, 430070, P. R. China

**Keywords:** miR-22, poly(I:C), type I interferon, inflammatory cytokines, MAVS

## Abstract

MicroRNAs (miRNAs) are small non-coding RNAs that play important roles in regulating the host immune response. Here we found that miR-22 is induced in glial cells upon stimulation with poly(I:C). Overexpression of miR-22 in the cultured cells resulted in decreased activity of interferon regulatory factor-3 and nuclear factor-kappa B, which in turn led to reduced expression of interferon-β and inflammatory cytokines, including tumor necrosis factor-α, interleukin-1β, interleukin-6, and chemokine (C-C motif) ligand 5, upon stimulation with poly(I:C), whereas knockdown of miR-22 had the opposite effect. We used a combination of bioinformatics and experimental techniques to demonstrate that mitochondrial antiviral signaling protein (MAVS), which positively regulates type I interferon production, is a novel target of miR-22. Overexpression of miR-22 decreased the activity of a luciferase reporter containing the MAVS 3′-untranslated region and led to decreased MAVS mRNA and protein levels. In contrast, ectopic expression of miR-22 inhibitor led to elevated MAVS expression. Collectively, our results demonstrate that miR-22 negatively regulates poly(I:C)-induced production of type I interferon and inflammatory cytokines via targeting MAVS.

## INTRODUCTION

Glial cells are the major central nervous system-resident cells, and represent critical effectors of central nervous system inflammation [1, 2]. Any disruption in the normal function of glial cells may have drastic consequences on brain function [3, 4]. Neuroinflammation is initiated upon recognition of the invading pathogen(s). Germ-line pathogen recognition receptors recognize pathogen-associated molecular patterns and initiate a host immune response against the invaders [5, 6].

Polyinosinic-polycytidylic acid (poly(I:C)) is a double-stranded RNA immune stimulant that is recognized by pathogen recognition receptors, and results in the production and release of several inflammatory cytokines [7]. In the central nervous system, glial cells can recognize poly(I:C), resulting in marked gliosis [8, 9]. Glial cells recognize poly(I:C) in two different ways [10]. In one way, poly(I:C) is internalized by endocytosis and recognized by toll-like receptor 3. In another way, poly(I:C) is recognized by melanoma differentiation-associated gene 5 (MDA-5) and retinoic acid inducible gene I [10]. Stimulation of toll-like receptor 3 by naked poly(I:C) results in nuclear factor-kappa B (NF-κB) activation, which in turn leads to production of inflammatory cytokines such as tumor necrosis factor (TNF)-α, chemokine (C-C motif) ligand 5 (CCL5), and interleukin (IL)-8 [11, 12]. On the other hand, expression of both retinoic acid inducible gene I and MDA-5 is increased in poly(I:C)-treated glial cells, but only MDA-5 is believed to play a central role in its recognition and the subsequent immune activation [13, 14]. Following recognition of poly(I:C) by MDA-5, MDA-5 associates with mitochondrial antiviral signaling protein (MAVS), and this interaction is tightly regulated. The MDA-5/MAVS complex then recruits both the IKKε/TBK1 and IKK α/β/γ complexes, which subsequently activate interferon regulatory factor 3 (IRF3) and NF-κB pathways [15–18].

MicroRNAs (miRNAs) are critical regulators of gene expression that utilize sequence complementarity to bind to and decrease the stability or translation efficiency of target mRNAs [19]. Recent studies have revealed that miRNAs participate in various biological processes such as organogenesis, cellular proliferation and differentiation, apoptosis, innate and adaptive immunity, inflammation, and tumorigenesis [20–25]. Accumulating evidence also suggests a decisive role for miRNAs in neuroinflammation. For instance, miR-155 targets suppressor of cytokine signaling 1 and modulates cytokine production in microglia [26], and it also negatively regulates blood-brain barrier function [27]. Recently, miR-200b and miR-210 were reported to reduce neuroinflammation [28, 29]. Critical roles for miR-181 and miR-146 in astrocyte-mediated inflammation have also been described [30, 31]. We have also previously reported a role for miR-206 in lipopolysaccharide-mediated inflammatory cytokine production in astrocytes [32]. Hence, several miRNAs mediate neuroinflammation in glial cells. However, the details of miRNA-mediated regulation of neuroinflammation remain unclear.

In the present study, we investigated that miR-22 is a negative regulator of type I interferon and inflammatory cytokine production in poly(I:C)-treated human glial cells. To the best of our knowledge, we demonstrated for the first time that the suppressive functions of miR-22 are achieved through targeting MAVS.

## RESULTS

### miR-22 level is upregulated in poly(I:C)-treated cells

**Figure 1:**
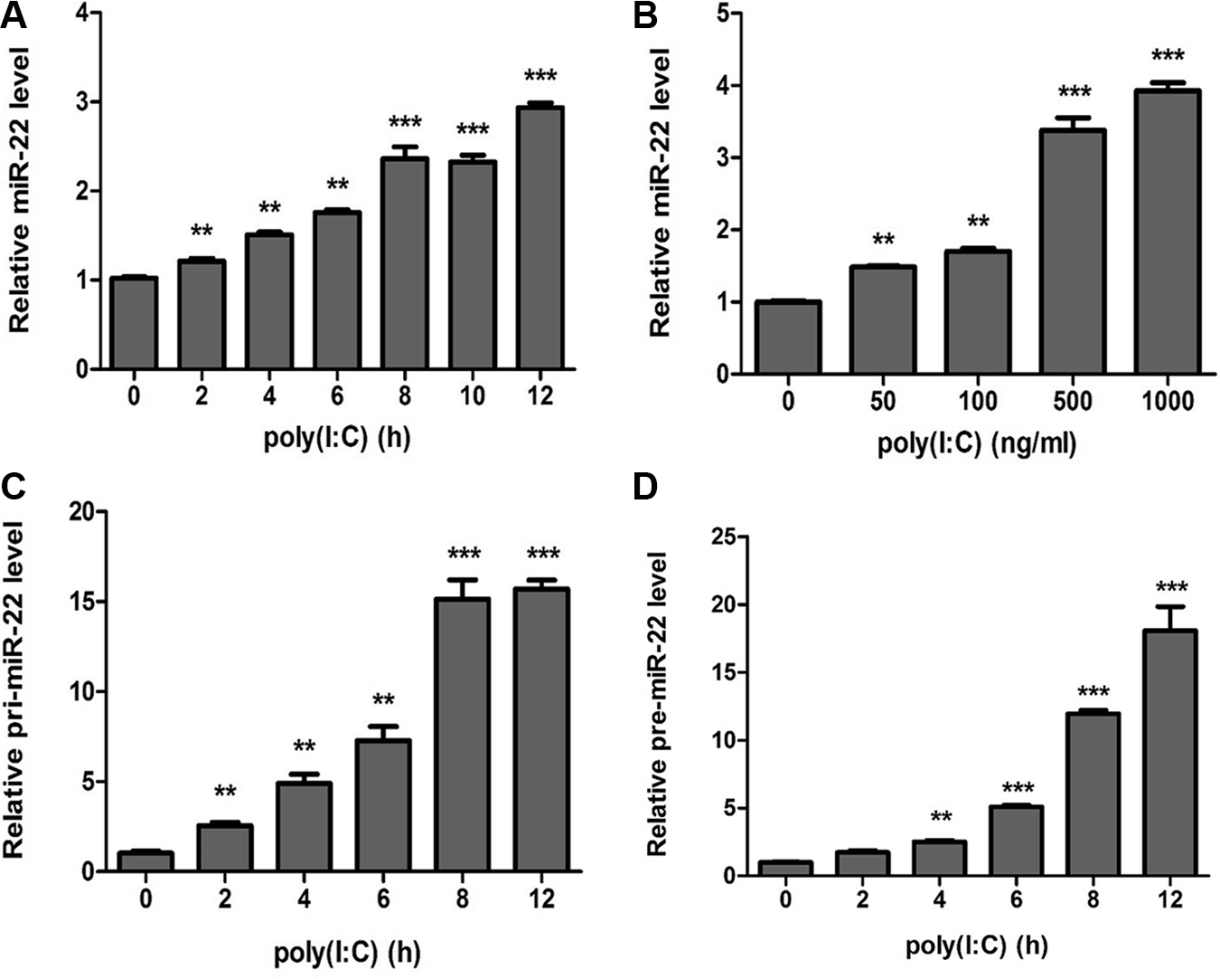
miR-22 is upregulated in poly(I:C)-treated glial cells (**A** and **B**) Human U251 cells were transfected with 100 ng/ ml poly(I:C) for different periods (A) or with different concentrations for 6 h (B), and miR-22 level was determined with quantitative real-time PCR. (**C** and **D**) U251 cells were transfected with 100 ng/ml poly(I:C) for different periods, and the level of each of pri-miR-22 (C) and pre-miR-22 (D) was detected with quantitative real-time PCR. All data are representative of at least three independent experiments. (**p < 0.01; ***p < 0.001).

Poly(I:C) induces increased expression of several miRNAs that control immune responses [33–35]. Of these, we identified miR-22 as a potentially neuroprotective miRNA based on its predicted regulation of targets implicated in immune responses. To delineate the kinetics of miR-22 in poly(I:C)-mediated immune responses, the expression of miR-22 was determined in poly(I:C)-treated U251 cells. The results revealed that miR-22 expression was significantly up-regulated in a time- (Figure 1A) and dose-dependent manner (Figure 1B). Similar to the results for mature miR-22, primary miR-22 transcripts (pri-miR-22) and miR-22 precursors (pre-miR-22) were also found to be up-regulated in poly(I:C)-stimulated U251 cells (Figure 1C and 1D). We also investigated the expression of miR-22 in poly(I:C)-treated SH-SY5Y cells (Supplementary Figure S1A and S1B), and the results were concordant with poly(I:C)-stimulated U251 cells. These findings suggest that miR-22 expression is up-regulated in poly(I:C)-treated cells.

### miR-22 reduces poly(I:C)-triggered type I interferon and inflammatory cytokine production

**Figure 2:**
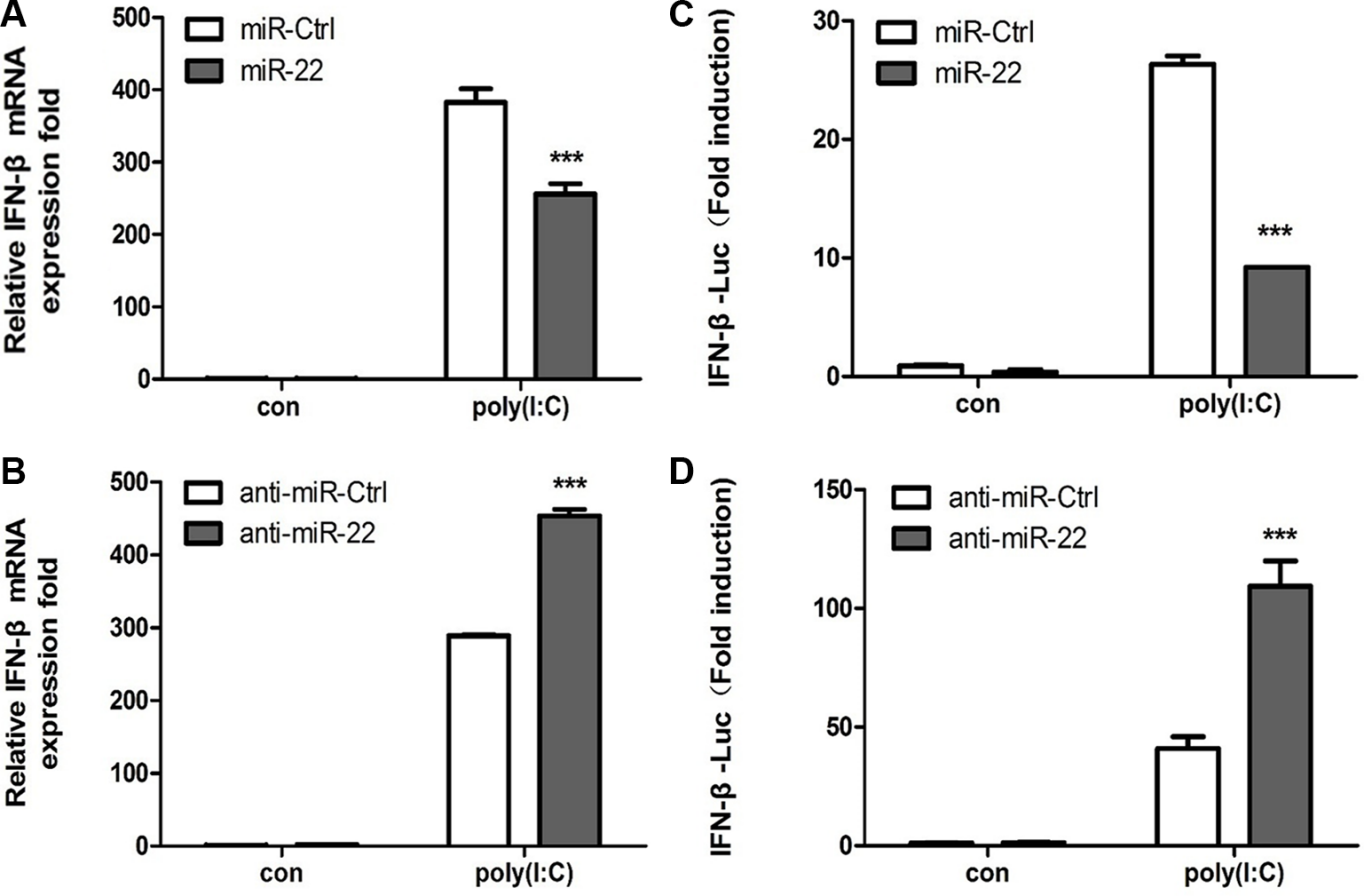
miR-22 negatively regulates poly(I:C)-triggered type I interferon production in human U251 cells (**A** and **B**) U251 cells were transfected with miR-22 mimics (A), miR-22 inhibitors (B), or the corresponding control oligo (final concentration, 50 nM) for 24 h and then transfected with 100 ng/ml poly(I:C) for 8 h. IFN-β mRNA levels were determined with quantitative real-time PCR and normalized to the expression of β-actin in each sample. (**C** and **D**) miR-22 mimics (C), miR-22 inhibitors (D), or the corresponding control oligo (final concentration, 50 nM) were co-transfected with IFN-β-Luc as well as pRL-TK (internal control). After 24 h, the cells were transfected with 100 ng/ml poly(I:C). Luciferase activity was measured 8 h later, and *Renilla reniformis* luciferase activity was normalized to the firefly luciferase activity. (***p < 0.001). All data are representative of at least three independent experiments.

Stimulation of cells with poly(I:C) results in a type I interferon immune response along with the production of other inflammatory cytokines [3, 36]. To identify whether poly(I:C)-induced miR-22 mediates poly(I:C)-triggered immune responses in U251 cells, we investigated the role of miR-22 in type I interferon production following stimulation of U251 cells with poly(I:C). Quantitative real-time PCR results revealed that overexpression of miR-22 significantly decreased poly(I:C)-triggered IFN-β production (Figure 2A), whereas inhibition of miR-22 expression increased poly(I:C)-triggered IFN-β production (Figure 2B). We next investigated the function of miR- 22 in IFN-β promoter activation. U251 cells were co- transfected with miR-22 mimics and the IFN-β luciferase reporter plasmids together with the internal control plasmid pRL-TK and then transfected with poly(I:C). Consistent with the effect of miR-22 on IFN-β production, miR-22 significantly suppressed IFN-β promoter activity in response to stimulation with poly(I:C) (Figure 2C). In contrast, inhibition of endogenous miR-22 expression enhanced IFN-β promoter activity (Figure 2D).

**Figure 3:**
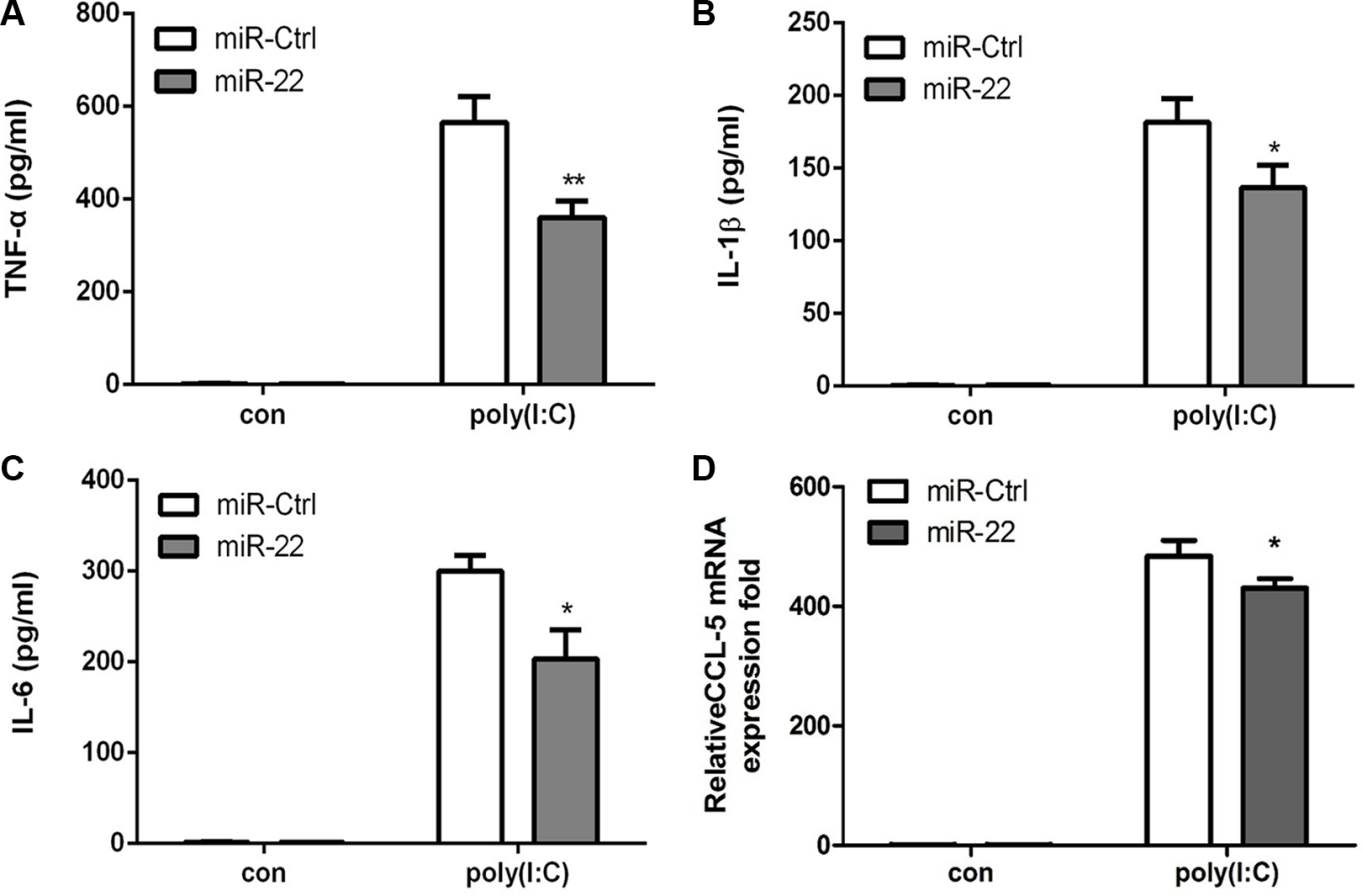
miR-22 suppresses poly(I:C)-triggered production of inflammatory cytokines (**A–D**) U251 cells were transfected with miR-22 mimics or control miRNA (final concentration, 50 nM) for 24 h and then transfected with 100ng/ml poly(I:C) for 8 h. The protein levels of TNF-α, IL-1β, and IL6 were analyzed by ELISA. Data represent means ± SD from three independent experiments performed in duplicate. (*p < 0.05; **p < 0.01). CCL5 mRNA levels were determined with quantitative real-time PCR and normalized to the expression of β-actin in each sample. All data are representative of at least three independent experiments. (*p < 0.05).

**Figure 4:**
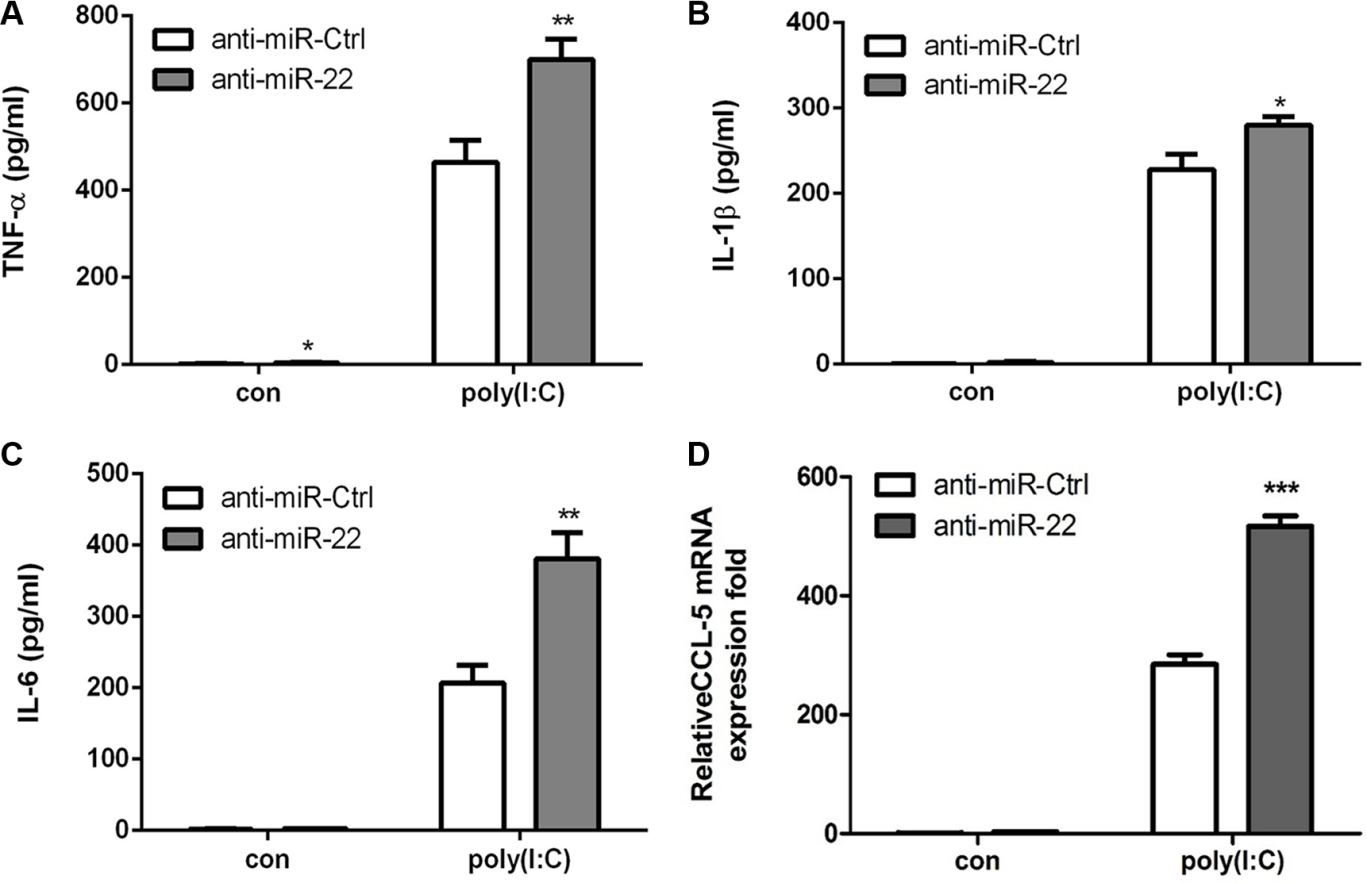
Inhibition of miR-22 increases poly(I:C)-triggered production of inflammatory cytokines (**A–D**) U251 cells were transfected with miR-22 inhibitors or control oligos (final concentration, 50 nM) for 24 h and then transfected with 100 ng/ml poly(I:C) for 8 h. The protein levels of TNF-α, IL-1β, and IL6 were analyzed by ELISA. Data represent means ± SD from three independent experiments performed in duplicate. (*p < 0.05; **p < 0.01). CCL5 mRNA levels were determined with quantitative real-time PCR and Zormalized to the expression of β-actin in each sample. All data are representative of at least three independent experiments. (***p < 0.001).

Next, the effects of miR-22 on related inflammatory cytokines were examined. In agreement with previous results, miR-22 overexpression reduced poly(I:C)-triggered production of TNF-α, IL-1β, IL6, and CCL5 (Figure 3A– 3D), whereas inhibition of miR-22 expression resulted in increased production of inflammatory cytokines (Figure 4A–4D). Taken together, these data strongly demonstrate that miR-22 reduces the production of type I interferon and inflammatory cytokines in poly(I:C)-treated cells.

### miR-22 targets human MAVS

**Figure 5:**
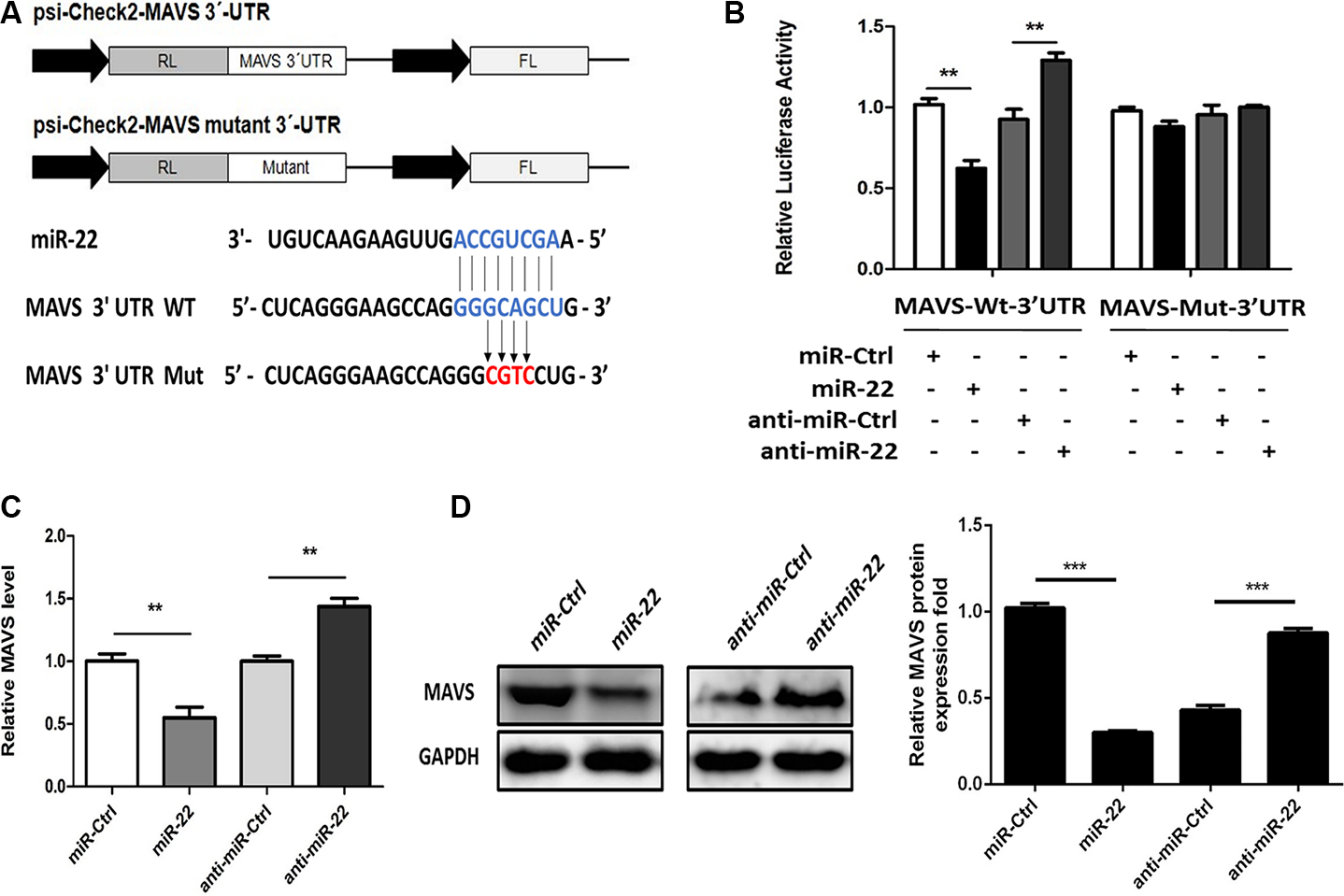
miR-22 targets human MAVS (**A**) The predicted miR-22 target sequence in the 3′-UTR of MAVS was downloaded from TargetScan. The mutant construct that contains four base pair mutations is shown below. The upper panel is the schematic diagram showing dual-luciferase reporter constructs harboring 3′-UTR of MAVS. (**B**) 293T cells were co-transfected with miR-22 mimics, miR-22 inhibitors, or the corresponding control oligo (final concentration, 50 nM) together with a wild-type (Wt) or mutated (Mut) MAVS 3′- UTR dual-luciferase reporter plasmid, and *Renilla* luciferase activity was measured and normalized to firefly luciferase activity after 24 h. (**C** and **D**) U251 cells were transfected with miR-22 mimics, miR-22 inhibitors, or the corresponding control oligo (final concentration, 50 nM), and then MAVS mRNA (C) and protein levels (D) were determined after 48 h with quantitative real-time PCR and immunoblotting, respectively. Protein levels were quantified with immunoblot scanning and normalized to the amount of GAPDH expression. All data are representative of at least three independent experiments. (**p < 0.01; ***p < 0.001).

We used the publically available miRNA target-prediction algorithms TargetScan, Pictar, PITA, miRBase, and RNAhybrid to identify miR-22 targets with potential relevance to control of type I interferon and inflammatory cytokine production. Among them, we selected MAVS as a potential target for miR-22. The crucial role of MAVS in the host innate immune response is well established [15, 16]. Both retinoic acid–inducible gene I and MDA- 5 recognize viral RNA and signal through a common adaptor molecule, i.e., MAVS, to activate the downstream IRF3 and NF-κB signaling pathways [36]. Therefore, we hypothesized that miR-22-mediated reduction of type I interferon and inflammatory cytokines may achieve through targeting MAVS. Our preliminary results using TargetScan, Pictar, PITA, miRBase, and RNAhybrid showed that MAVS had potential seed matches for miR-22 and thus, was further investigated. The predicted miR-22 target sequences in the 3′-UTR of MAVS are shown in Figure 5A. The MAVS 3′-UTR containing a potential seed match region was cloned into the dual-luciferase reporter plasmid (Figure 5A), and 293T cells were co-transfected with this reporter plasmid and miR-22 mimics or inhibitors. The luciferase signal decreased significantly following transfection of miR- 22 mimics. By contrast, luciferase activity increased following treatment with miR-22 inhibitors (Figure 5B). To confirm that this reduction in luciferase activity was indeed due to interaction of miR-22 with the 3′-UTR of MAVS, a mutant dual luciferase reporter containing four base pair mutations in the seed region was also co- transfected into 293T cells together with miR-22 mimics or inhibitors. As expected, no significant effect of either miR-22 mimics or inhibitors was observed (Figure 5B). To further probe the impact of the interaction between miR-22 and the MAVS 3′-UTR, expression of endogenous MAVS was measured in U251 cells treated with miR-22 mimics or inhibitors. As predicted, ectopic expression of miR-22 resulted in marked reduction in MAVS both at the transcriptional and post-transcriptional levels, whereas miR-22 inhibitors increased the expression of MAVS (Figure 5C and 5D). Thus, these results suggest that MAVS is a direct target of miR-22, and its expression is repressed by miR-22.

### MAVS expression is downregulated in poly(I:C)-treated cells

**Figure 6:**
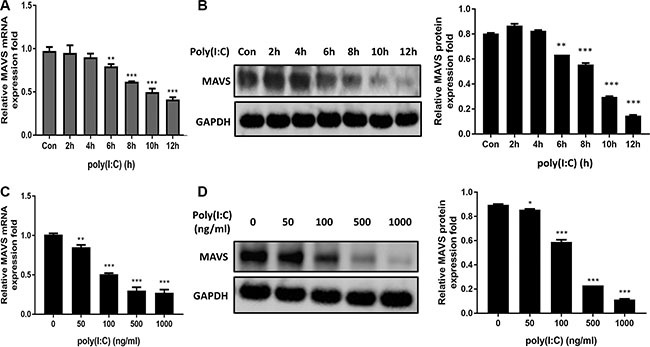
MAVS expression is downregulated in poly(I:C)-treated glial cells (**A** and **B**) Human U251 cells were transfected with 100ng/ml poly(I:C) for different periods, and MAVS mRNA (A) and protein (B) level was determined with quantitative real-time PCR and immunoblotting, respectively. (**C** and **D**) U251 cells were transfected with poly(I:C) with different concentrations for 6h, and and MAVS mRNA (C) and protein (D) level was determined with quantitative real-time PCR and immunoblotting, respectively. Data represent means ± SD from three independent experiments. *p < 0.05; **p < 0.01; ***p < 0.001. Protein levels were quantified with immunoblot scanning and normalized to the amount of GAPDH expression.

To study the effect of poly(I:C) on MAVS, time-dependent and dose-dependent expression pattern of MAVS mRNA (Figure 6A and 6C) and protein (Figure 6B and 6D) in U251 cells following poly(I:C) treatment was studied. Significant downregulation of MAVS mRNA and protein levels were observed in poly(I:C)-treated cells. Furthermore, MAVS mRNA and protein expression levels were also determined in poly(I:C)-stimulated SH-SY5Y cells (Supplementary Figure S1C and S1D). The results were similar to those as were observed in U251 cells. Thus, these data demonstrate that MAVS expression is downregulated upon poly(I:C) treatment.

### miR-22 reduces poly(I:C)-triggered IFN-β and inflammatory cytokine production via targeting MAVS

**Figure 7:**
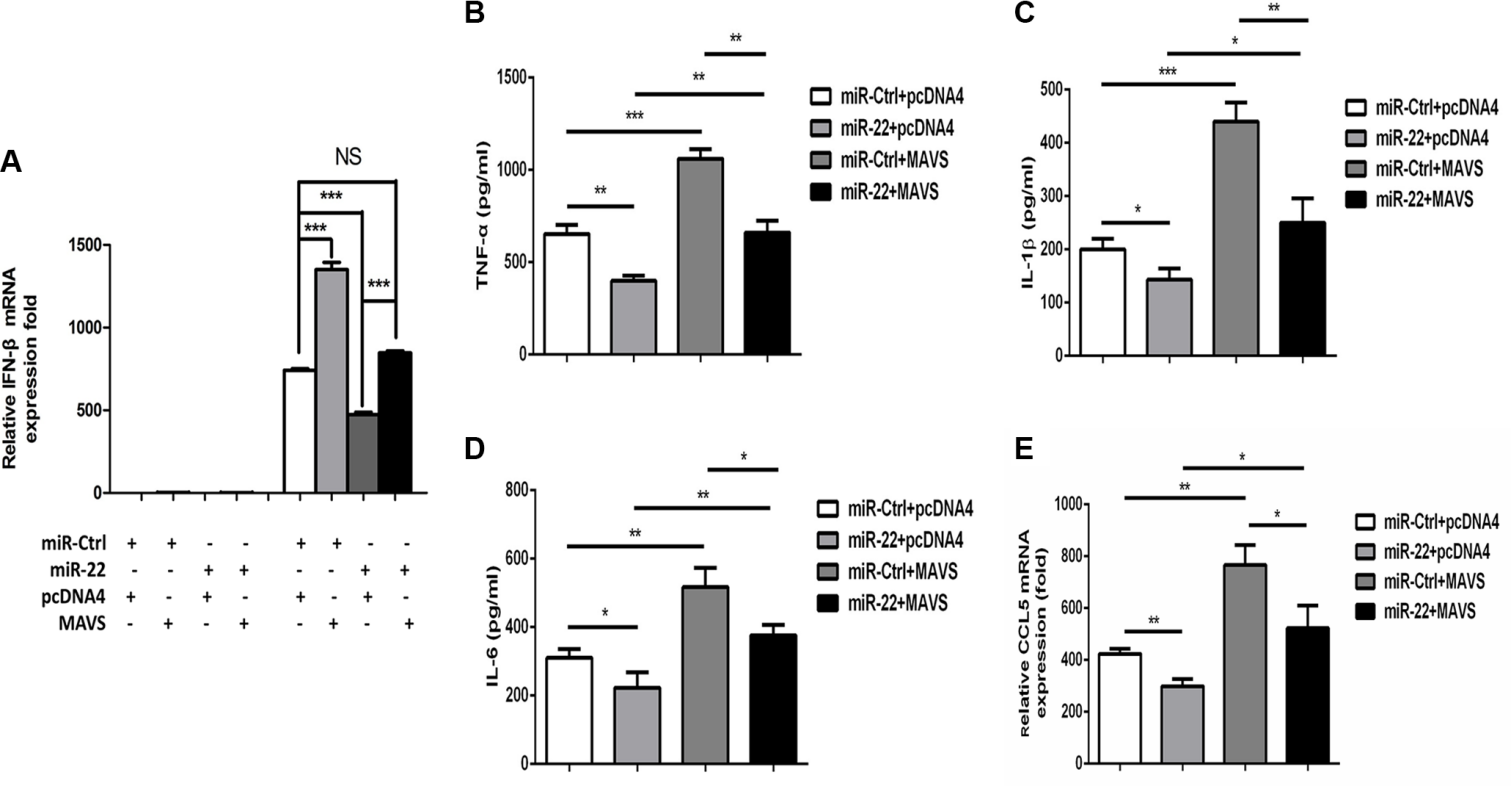
Overexpression of MAVS enhances the poly(I:C)-triggered production of IFN-β and inflammatory cytokines (**A**) U251 cells were co-transfected with miR-22 mimics or control mimics and pCDNA4 (200 ng) or pCDNA4-MAVS (200 ng) for 24 h and either left untransfected or transfected with 100 ng/ml poly(I:C). After 8 h, total RNA was extracted, and IFN-β level was determined with quantitative real-time PCR. (**B–E**) U251 cells were co-transfected with miR-22 mimics or control mimics and pCDNA4 (200 ng) or pCDNA4-MAVS (200ng) for 24 h and either left untransfected or transfected with 100ng/ml poly(I:C) for 8 h. The protein levels of TNF-α, IL-1β, and IL6 were analyzed by ELISA. Data represent means ± SD from three independent experiments performed in duplicate. (*p < 0.05; **p < 0.01). CCL5 mRNA levels were determined with quantitative real-time PCR and normalized to the expression of β-actin in each sample. All data are representative of at least three independent experiments. (*p < 0.05; **p < 0.01).

Overexpression of MAVS is sufficient to activate the IRF3/7 and NF-κB pathways to induce type I interferon and inflammatory cytokines production, respectively [15]. We therefore determined whether the MAVS expression plasmid could rescue the inhibition of type I interferon and inflammatory cytokine production mediated by miR- 22. U251 cells were co-transfected with miR-22 mimics and the MAVS expression plasmid, and then transfected with poly(I:C). We found that overexpression of MAVS fully rescued the suppression of type I interferon (Figure 7A) and inflammatory cytokines (Figure 7B–7E) observed upon miR-22 overexpression.

**Figure 8:**
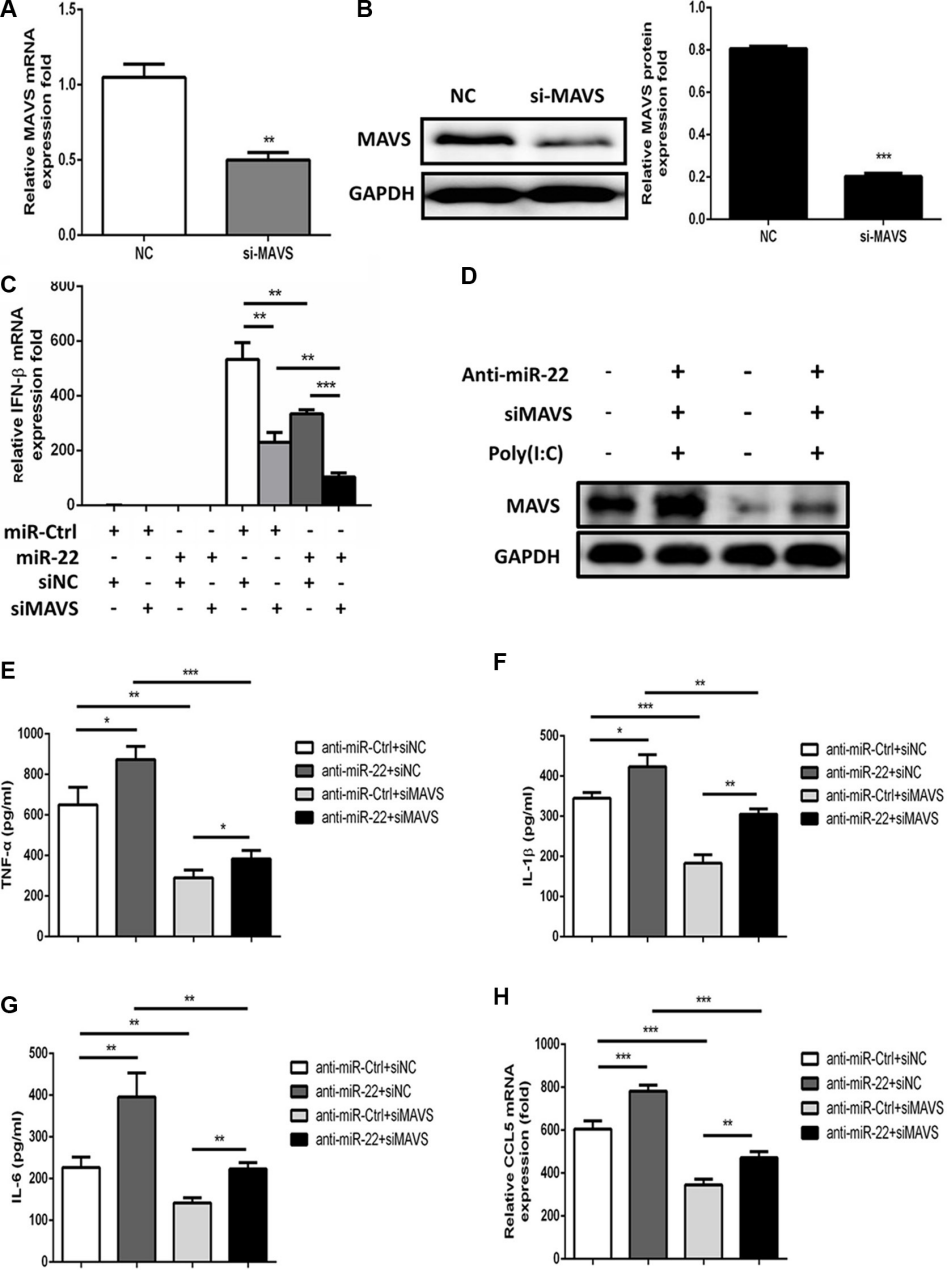
Silencing of MAVS reduces the poly(I:C)-triggered production of IFN-β and inflammatory cytokines (**A** and **B**) U251 cells were transfected with siMAVS or nonspecific control siRNA (final concentration, 50 nM) for 24 h, and then MAVS mRNA (A) and protein (B) levels were measured with quantitative real-time PCR and immunoblotting, respectively. Protein levels were quantified with immunoblot scanning and normalized to the amount of GAPDH expression. (**C**) U251 cells were co-transfected with miR-22 inhibitors or control oligos and siMAVS or non-specific control siRNA (final concentration, 50 nM) for 24 h, and then either left untransfected or transfected with 100 ng/ml poly(I:C). After 8 h, total RNA was extracted, and IFN-β level was determined with quantitative real-time PCR. (**D**–**H**) U251 cells were co-transfected with miR-22 inhibitors or control oligos and siMAVS or non-specific control siRNA (final concentration, 50 nM) for 24 h, and then either left untransfected or transfected with 100 ng/ml poly(I:C) for 8 h. (D) MAVS protein levels were determined by immunoblotting. Protein levels were normalized to the amount of GAPDH expression. (E–H) The protein levels of TNF-α, IL-1β, and IL6 were analyzed by ELISA. Data represent means ± SD from three independent experiments performed in duplicate. (*p < 0.05; **p < 0.01; ***p < 0.001). CCL5 mRNA levels were determined with quantitative real-time PCR and normalized to the expression of β-actin in each sample. All data are representative of at least three independent experiments. (**p < 0.01; ***p < 0.001).

To further examine whether the observed effects of miR-22 on type I interferon and inflammatory cytokines in response to poly(I:C) were, at least partially, mediated through MAVS, we analyzed the effects of silencing of MAVS expression by siRNA in U251 cells. The cells were co-transfected with miR-22 inhibitors and MAVS-specific siRNA, and then transfected with poly(I:C). First, we confirmed that the siRNA significantly inhibited MAVS in U251 cells at both the mRNA and protein levels (Figure 8A and 8B). Knockdown of MAVS significantly decreased the expression level of IFN-β and inflammatory cytokines, which means that MAVS silencing produces effects similar to those of miR-22 overexpression (Figure 8C and 8D–H). Thus, these data demonstrate that reduction of poly(I:C)-triggered IFN-β and inflammatory cytokines by miR-22 was achieved through MAVS.

### miR-22 suppresses the interferon pathway downstream of MAVS

**Figure 9:**
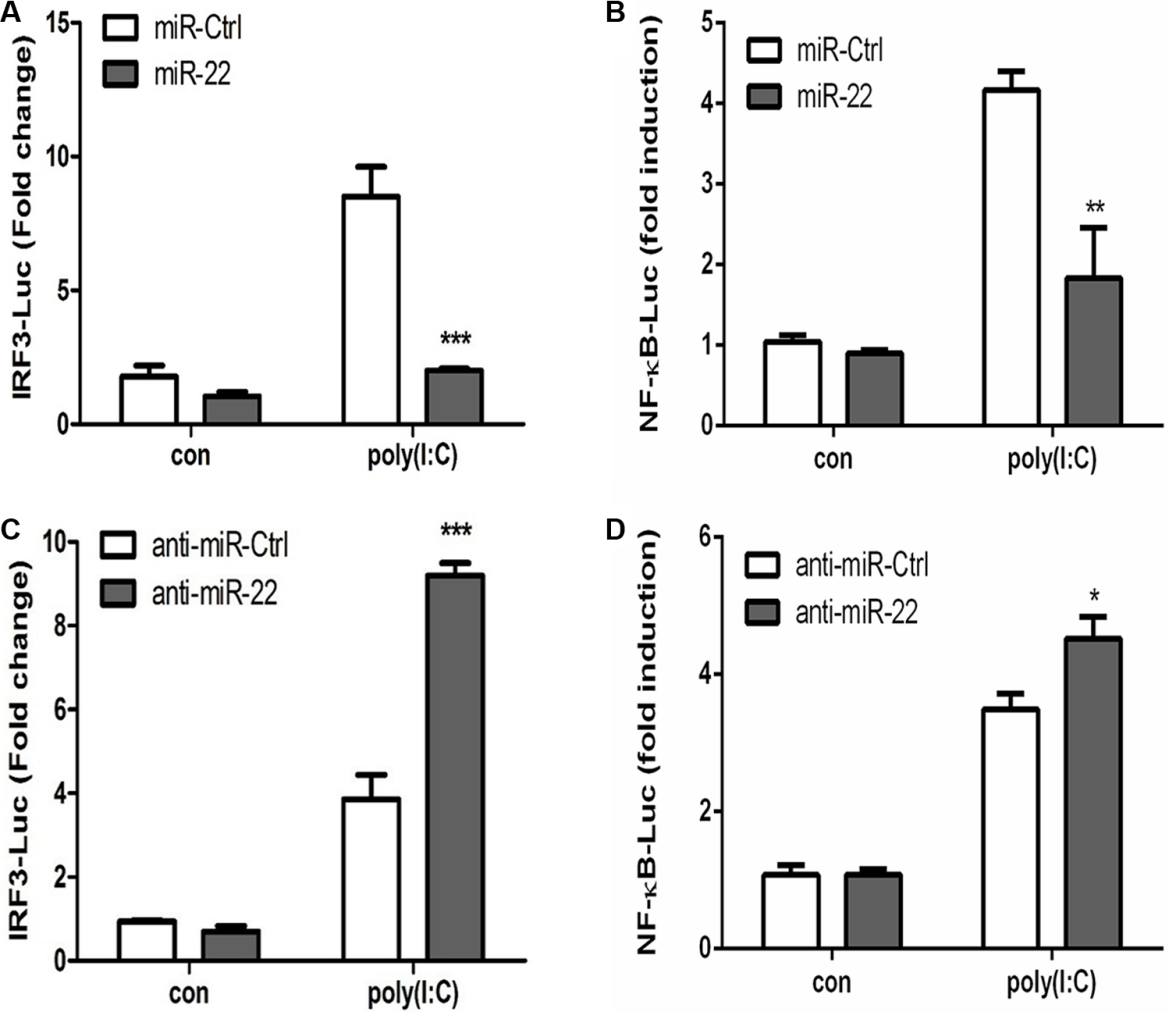
miR-22 negatively regulates the activity of transcriptional factors IRF3 and NF-κB (**A** and **B**) miR-22 mimics or the corresponding control oligo (final concentration, 50 nM) were co-transfected with IRF3-Luc or NF-κB-Luc, as well as pRL-TK (internal control). After 24 h, the cells were transfected with 100 ng/ml poly(I:C). Luciferase activity was measured 8 h later, and the *Renilla reniformis* luciferase activity was normalized to firefly luciferase activity. (**C** and **D**) miR-22 inhibitors or the corresponding control oligo (final concentration, 50 nM) were co-transfected with IRF3-Luc or NF-κB-Luc, as well as pRL-TK (internal control). After 24 h, the cells were transfected with 100 ng/ml poly(I:C). Luciferase activity was measured 8 h later, and the *Renilla reniformis* luciferase activity was normalized to firefly luciferase activity. *p < 0.05; **p < 0.01; ***p < 0.001.

**Figure 10:**
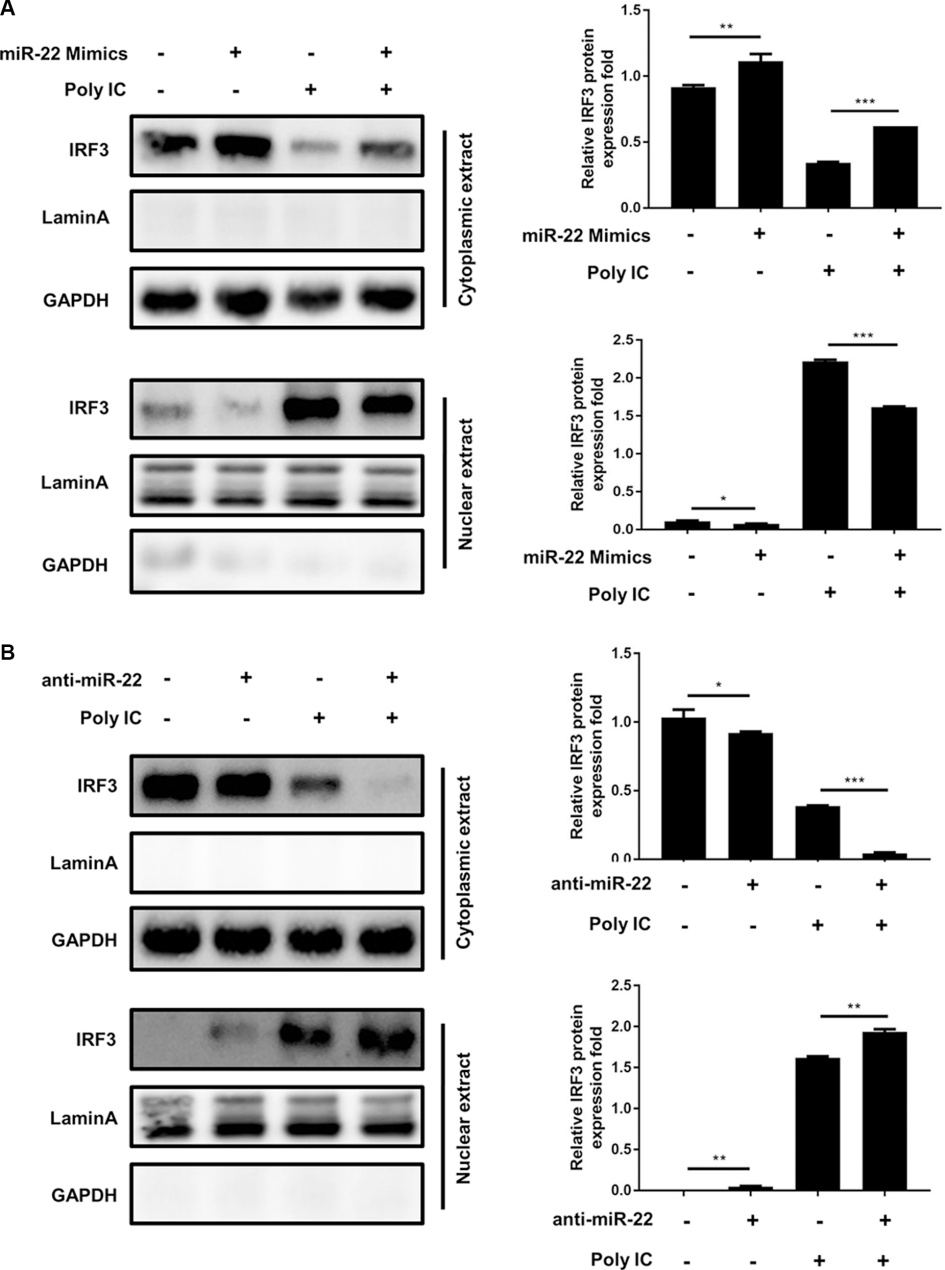
miR-22 suppresses the nuclear translocation of IRF3 (**A** and **B**) U251 cells were transfected with miR-22 mimics (A), miR-22 inhibitors (B), or the corresponding control oligo (final concentration, 50 nM) for 24 h and then transfected with poly(I:C) for another 8 h. The cytosolic extracts (upper panel) and nuclear extracts (lower panel) were isolated and subjected to immunoblotting with antibodies against IRF3, LaminA, and GAPDH. LaminA was used as a marker for nuclei. GAPDH and LaminA were used as the loading control. Protein levels were quantified with immunoblot scanning and normalized to the amount of GAPDH or LaminA expressions. *p < 0.05; **p < 0.01; ***p < 0.001.

**Figure 11:**
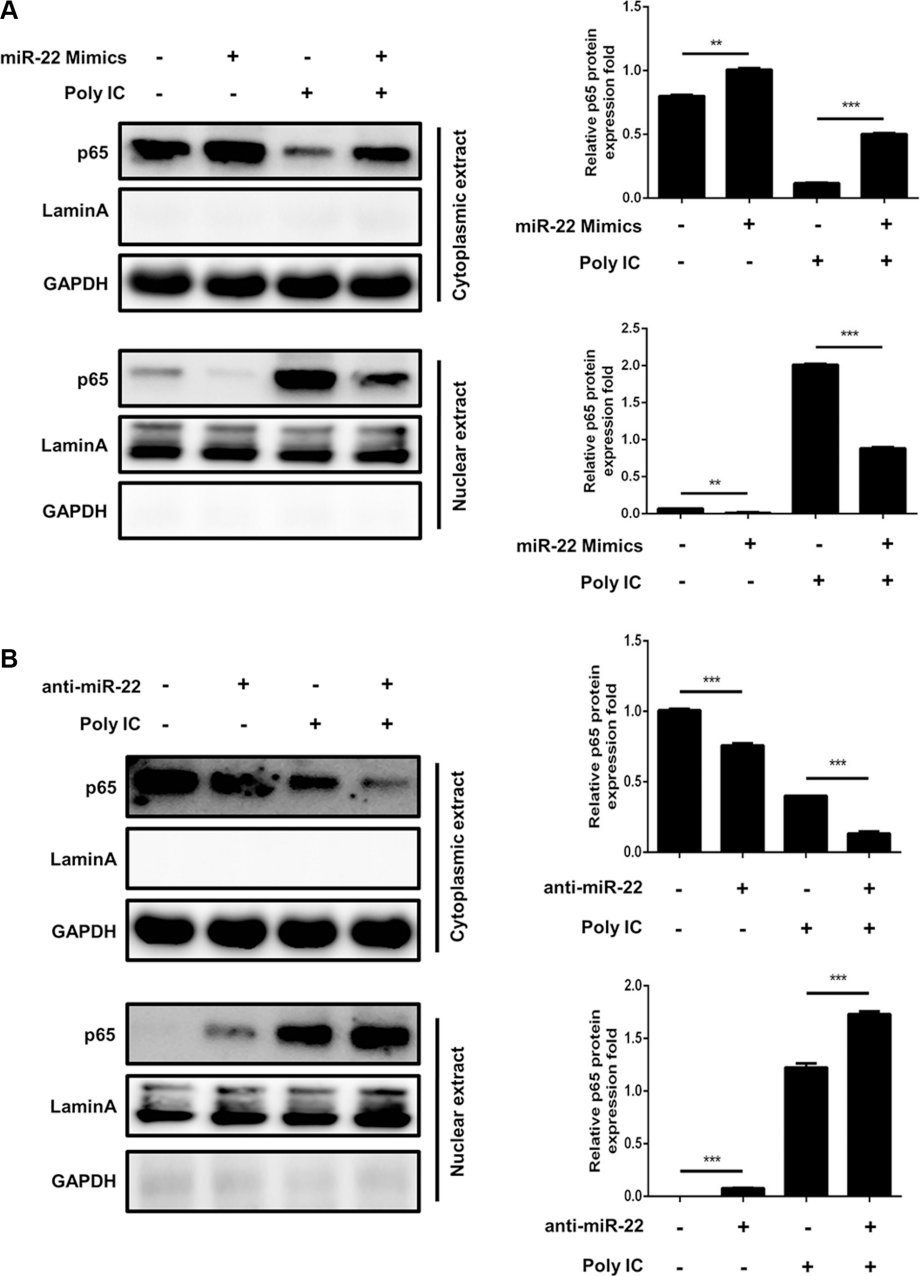
miR-22 inhibits the translocation of NF-κB from the cytoplasm to the nucleus (**A** and **B**) U251 cells were transfected with miR-22 mimics (A), miR-22 inhibitors (B), or the corresponding control oligo (final concentration, 50 nM) for 24 h and then transfected with poly(I:C) for another 8 h. The cytosolic extracts (upper panel) and nuclear extracts (lower panel) were isolated and subjected to immunoblotting with antibodies against NF-κB (p65), LaminA, and GAPDH. LaminA was used as a marker for nuclei. GAPDH and LaminA were used as the loading control. Protein levels were quantified with immunoblot scanning and normalized to the amount of GAPDH or LaminA expressions. **p < 0.01; ***p < 0.001.

Activated MAVS can recruit TNF receptor–associated factor family proteins, which leads to production of type I interferon and inflammatory cytokines through activation of the IRF3 and NF-κB [16, 17]. To further define the mechanisms by which miR-22 regulates the production of type I interferon and inflammatory cytokines, the impact of miR-22 on activation of IRF3 and NF-κB was determined in U251 cells treated with poly(I:C). First, we investigated the effect of miR-22 on the activity of IRF3 and NF-κB. U251 cells were co-transfected with miR-22 mimics and the luciferase reporter plasmids harboring the IRF3 or NF-κB binding sites together with the internal control plasmid pRL- TK, and then transfected with poly(I:C). Consistent with the effect of miR-22 on IFN-β promoter activity, miR-22 significantly suppressed the activity of IRF3 and NF-κB upon stimulation with poly(I:C) (Figure 9A and 9B). In contrast, inhibition of endogenous miR-22 expression enhanced the activities of both transcriptional factors (Figure 9C and 9D). It has been well established that translocation of NF-κB from the cytoplasm to the nucleus is a key determinant of NF-κB activation [37, 38]. Therefore, it was of interest to evaluate the effect of miR- 22 on NF-κB and IRF3 activation in poly(I:C)-treated cells. Nuclear translocation of NF-κB (p65) and IRF3 was detected with immunoblotting. Transfection of miR- 22 mimics inhibited the translocation of IRF3 and NF-κB from the cytoplasm to the nucleus (Figure 10A and Figure 11A). In contrast, treatment of cells with miR-22 inhibitors significantly increased the nuclear translocation of IRF3 and NF-κB in poly(I:C)-stimulated U251 cells (Figure 10B and Figure 11B). Taken together, these findings demonstrate that miR-22 appears to regulate the signaling pathway downstream of MAVS.

### Japanese encephalitis virus (JEV) infection upregulates miR-22 expression and downregulates MAVS expression

**Figure 12:**
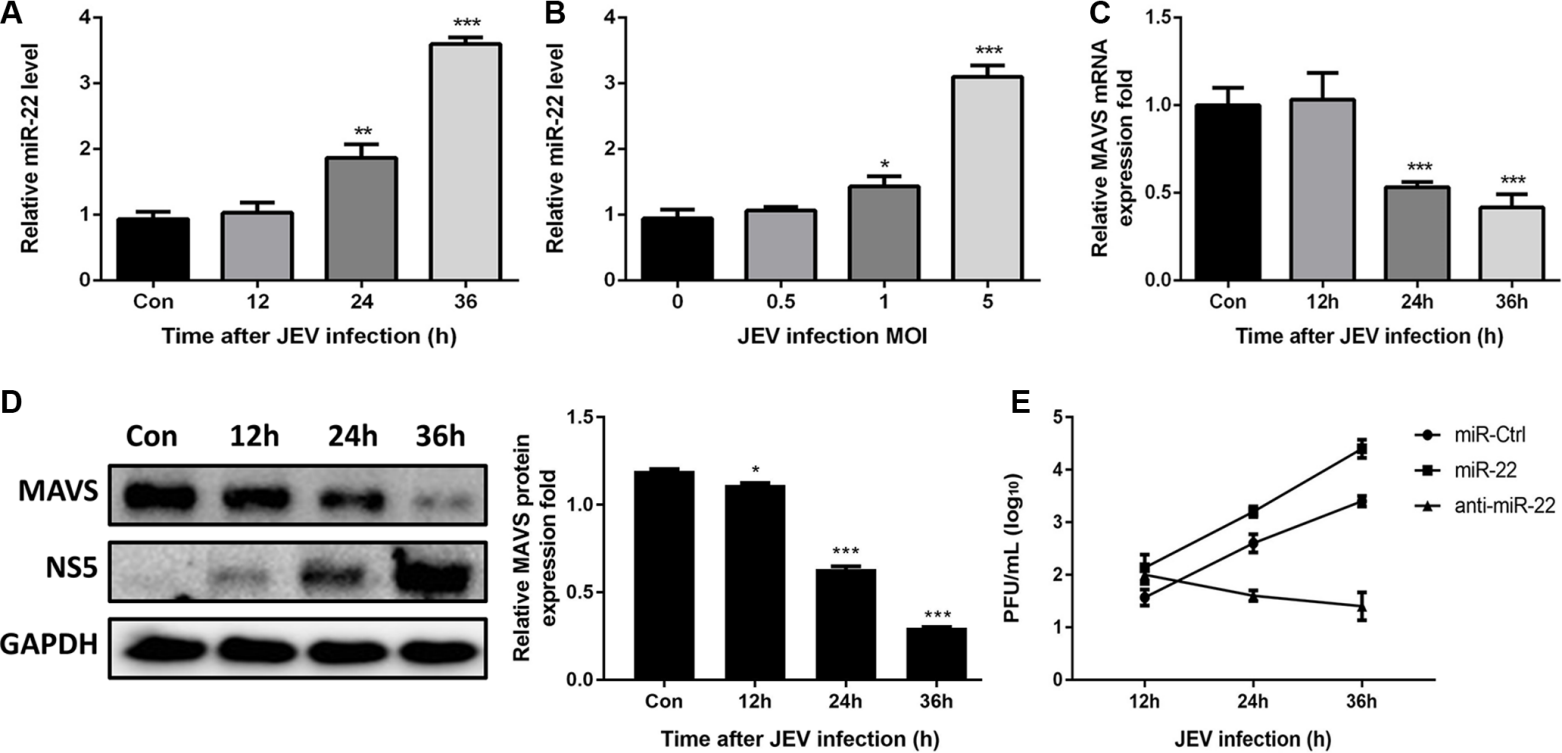
JEV infection upregulates miR-22 expression and downregulates MAVS expression (**A** and **B**) U251 cells were infected with JEV at MOI of 5 for the indicated times (A), or at indicated MOIs for 36 h (B). The levels of miR-22 were detected by quantitative real-time-PCR. (**C** and **D**) U251 cells were infected with JEV at MOI of 5 for the indicated times, and then MAVS mRNA (C) and protein (D) expression levels were determined by quantitative real-time PCR and immunoblotting, respectively. Data represent means ± SD from three independent experiments. *p < 0.05; **p < 0.01; ***p < 0.001. Protein levels were quantified with immunoblot scanning and normalized to the amount of GAPDH expression. (**E**) The transfected U251 cells were infected with JEV at MOI of 5. Cells were collected at the indicated time points, and titers of infectious virus in the culture supernatants were determined by plaque assay. The data represent three independent experiments with identical results.

Because poly(I:C) is used as analog of viral double-stranded RNA, we hypothesized that upregulation of miR-22 expression in glial cells would facilitate the replication of neurotropic viruses such as JEV which can replicate effectively in glial cells. To study the effect of JEV infection on miR-22 and MAVS, expression pattern of miR-22 and MAVS in U251 cells following JEV infection was studied. The results revealed that miR- 22 was significantly up-regulated in a time-dependent (Figure 12A) and dose-dependent manner (Figure 12B). Significant down-regulation of MAVS mRNA and protein levels at 12, 24, and 36 h post- infection was also observed (Figure 12C and 12D). Furthermore, we found that treatment of cells with miR-22-specific mimics enhanced the JEV replication, whereas miR-22-specific inhibitors exhibited an antiviral activity (Figure 12E). These findings suggest that JEV infection upregulates miR-22 expression and downregulates MAVS expression in glial cells.

## DISCUSSION

Innate immunity is a vital component of central nervous system, and entrusts mainly on resident glial cells [39]. Glial cells exhibits an array of receptors associated with innate immunity such as Toll like receptors, double-stranded RNA-dependent protein kinase, nucleotide-binding oligomerization domains, mannose receptors, scavenger receptors, and components of the complement system [40–44]. Inflammation in the central nervous system is characterized by marked activation of these glial cells [45]. Upon activation, glial cells are endowed with the ability to produce type I interferons, inflammatory cytokines, and chemokines [39, 46]. Multiple miRNAs can regulate human innate immune signaling pathways and mediate inflammatory responses. Recently, numerous miRNAs have been reported to play pivotal roles in neuroinflammation. For example, miR-155 modulates JEV–induced neuroinflammation by targeting Src homology 2–containing inositol phosphatase 1 [47]. Another miRNA, miR-29b, regulates JEV–induced microgliosis by targeting TNF-α–induced protein 3 [48]. Recently, we also reported the roles of miR-15b and miR-19b-3p in JEV-mediated neuroinflammation in glial cells by targeting ring finger protein 125 and 11, respectively [38, 49]. However, the role of miRNAs in reducing neuroinflammation is largely unknown. Here, we demonstrated that miR-22 is a negative regulator of poly(I:C)-induced neuroinflammation in human glial cells. Briefly, miR-22 inhibited MAVS expression and negatively regulated poly(I:C)-triggered production of type I interferon and inflammatory cytokines in U251 cells.

miR-22 has been found to be ubiquitously expressed in various tissues [50, 51]. Evolutionary clustering suggests that miR-22 is highly conserved in vertebrate evolution, indicating its functional importance in vertebrate species. It had been deduced from the statistical analysis of 3′-UTR in transcriptome that miR-22 is involved in the regulation of many target genes [52]. Previous studies have suggested that miR-22 functioned in multiple cellular processes such as proliferation, differentiation, apoptosis, senescence, and its deregulation is a hallmark of cancer [53–55]. However, the neuroprotective activity of miR-22 remains poorly understood. In the present study, we demonstrated that miR-22 was upregulated in U251 cells treated with poly(I:C) and repressed the production of type I interferon and inflammatory cytokines by targeting MAVS. Poly(I:C) activates MDA5 and its downstream mediator MAVS, which subsequently results in activation of IRF3 and NF-κB signaling pathways [10]. Interestingly, overexpression of miR-22 markedly inhibited the expression of MAVS in U251 cells. Also, our findings demonstrated that miR-22 inhibited the translocation of IRF3 and NF-κB from the cytoplasm to the nucleus, which in turn led to reduced activation of IFN-β and inflammatory cytokine genes. Knockdown of MAVS with siRNA induced the same effects on IFN-β and inflammatory cytokines levels as was observed following miR-22 overexpression, whereas overexpression of MAVS abrogated the miR-22–mediated inhibitory effects on IFN-β and inflammatory cytokine production in poly(I:C)-treated U251 cells. Taken together, these results indicate that poly(I:C)-induced miR-22 acts as a potent anti-neuroinflammatory miRNA in human glial cells.

Previously, it has been reported that MAVS expression could be downregulated by virus infection via ubiquitin-proteasome-dependent pathways and mRNA instability [56, 57]. However, in this study, we proposed that MAVS expression could be modulated via miR- 22, suggesting a new mechanism of miRNA-mediated downregulation of MAVS.

Many viruses can take advantage of host-encoded miRNAs to modulate the host innate immune response, resulting in increased viral replication. miR-146a negatively regulates vesicular stomatitis virus–triggered production of type I interferon by targeting TNF receptor–associated factor 6 and the kinases IRAK1 and IRAK2 in macrophages, thus promoting vesicular stomatitis virus replication [58]. miR-146a also promotes replication of other important viruses such as Hendra virus, dengue virus, and enterovirus 71 [59–61]. miR- 21 suppresses hepatitis C virus–triggered production of type I interferon by targeting MyD88 and IRAK1 in hepatocytes, which results in increased hepatitis C virus replication [62]. Because poly(I:C) is used as analog of viral double-stranded RNA and miR-22 represses poly(I:C)-triggered production of type I interferon and inflammatory cytokines, we predict that upregulation of miR-22 expression in glial cells would facilitate the replication of neurotropic viruses which can replicate effectively in glial cells. To confirm this prediction, we determined the expression pattern of miR-22 and MAVS in JEV-infected glial cells, and the results showed that JEV infection upregulated the expression pattern of miR-22 and downregulated the levels of MAVS. We also observed that treatment of cells with miR-22 mimics resulted in enhanced JEV replication, whereas inhibitors had the opposite results. From these findings, we propose that other neurotropic viruses may utilize the similar strategy to invade host immune response. To validate this prediction, further studies are needed.

## MATERIALS AND METHODS

### Cell culture and treatment

U251 cells (human astrocytoma cell line), SH-SY5Y cells (human neuroblastoma cell line), and HEK 293T cells (human embryonic kidney epithelial cells) were cultured and maintained in Dulbecco's Modified Eagle's Medium that was supplemented with 10% fetal bovine serum, 100 U/ml penicillin, and 100 mg/ml streptomycin sulfate at 37°C in 5% CO_2_. U251 cells were plated in 12-well plates (1×10^5^ cells/well) and grown to 80% confluency. Non-adherent cells were removed by washing with non-supplemented DMEM prior to further treatment. Cells were subsequently transfected with RNAs and/or plasmids using Lipofectamine 2000 (Invitrogen). After 24 h, cells were transfected with poly(I:C) for another 12 h. For JEV infection experiments, cells were infected with JEV at multiplicity of infection (MOI) of 5 for indicated times.

### Reagents

Poly(I:C) was obtained from Sigma. Antibodies against IRF3 were obtained from Cell Signaling Technology. Antibodies against MAVS were purchased from Proteintech Technology. Antibodies against p65, glyceraldehyde-3-phosphate dehydrogenase (GAPDH), and LaminA were purchased from Abclonal Technology. Horseradish peroxidase–conjugated anti-mouse/rabbit secondary antibodies were from Boster. miR-22 mimics (double-stranded RNA oligonucleotides) and miR-22 inhibitors (single-stranded chemically modified oligonucleotides) from GenePharma were used for the overexpression and inhibition of miR-22 activity, respectively. Small interfering RNA (siRNA) oligonucleotide and control for MAVS were also purchased from GenePharma. Table 1 lists the sequences of the miR-22 mimics, miR-22 inhibitors, siMAVS oligonucleotides, and their controls.

**Table 1:** Sequences of RNAi oligonucleotides used in the present study

Name	Sequence 5′→3′
si-MAVS	UAGUUGAUCUCGCGGACGA-dTdT (sense)
	UCGUCCGCGAGAUCAACUA-dTdT (antisense)
control mimic	UUCUCCGAACGUGUCACGUTT (sense)
	ACGUGACACGUUCGGAGAATT (antisense)
miR-22 mimic	AAGCUGCCAGUUGAAGAACUGU (sense)
	AGUUCUUCAACUGGCAGCUUUU (antisense)
control inhibitor	CAGUACUUUUGUGUAGUACAA
miR-22 inhibitor	ACAGUUCUUCAACUGGCAGCUU

### microRNA target prediction

Putative microRNA target genes were identified using the microRNA databases (http://www.mirbase.org/) and target prediction tools PicTar (http://pictar.mdc-berlin.de/), TargetScan (http://www.targetscan.org/), PITA (https://omictools.com/pita-tool), and RNAhybrid (http://bibiserv.techfak.uni-bielefeld.de/rnahybrid/). The potential targets of miR-22 predicted in these databases were filtered on the basis of conserved and poorly conserved sites for miR-22, aggregate P_CT_ value, and potential relevance of predicted targets with inflammatory pathways. P_CT_ value is the probability of conserved targeting for highly conserved miRNAs.

### Constructs and plasmids

The psiCheck-2 dual-luciferase reporter vector (Promega) harboring the 3′-untranslated region (UTR) of MAVS was inserted into the *Xho*I and *Pme*I restriction sites at the 3′-end of *Renilla*, and was used to check the effect of miR-22 on *Renilla* luciferase activity. The 3′-UTR and the coding region of MAVS were amplified from U251 cell genomic DNA with specific primers and cloned into psiCheck-2 and pCDNA4, respectively (Life Technologies). The psiCheck-2 mutant MAVS 3′-UTR construct was generated by inducing point mutations with overlap-extension PCR method. Table 2 lists all primer sequences. All constructs were verified with sequencing.

**Table 2:** Sequences of the primers used in the present study

Primer	Sequence 5′→3′
miR-22 stem-loop primer	GTCGTATCCAGTGCAGGGTCCGAGGTATTCGCACTGGATACGACACAGTT
miR-22-F	AAGCTGCCAGTTGAAGAACTGT
Universal reverse primer	GTGCAGGGTCCGAGGT
pri-miR-22-F	AGGAGTAGAAGGCTCAAACA
pri-miR-22-R	AGGAGGGTCAAGAAGGAA
pre-miR-22-F	GGCGAGCCGCAGAGC
pre-miR-22-R	GGCGGGGCCGTTCTT
hCCL5-F	CTGTCATCCTCATTGCTACTGC
hCCL5-R	ATGTACTCCCGAACCCATTTCT
hIFN-β-F	TTCCCAGGGCTTACACCG
hIFN-β-R	TAACCCAAGTTCCCGAGT
hMAVS-3′-UTR-XhoI-F	ACGCTCGAGAGCCCAGCCTGAGACCGT
hMAVS-3′-UTR-PmeI-R	CGGTTTAAACCTGCACCCTTGACCACTGTG
Mutant-primer-2-R	CTGACACGACCCCCTGGC
Mutant-primer-3-F	AGCCAGGGGGTCGTGTC
hMAVS-KpnI-F	CGAGGTACC ATGCCGTTTGCTGAAGACAAGACC
hMAVS-XhoI-R	ACGCTCGAGGTGCAGACGCCGCCGGT

h: human.

### Dual-luciferase reporter assays

For the MAVS 3′-UTR luciferase reporter assay, 239T cells were co-transfected with 200 ng psiCheck-2 dual-luciferase plasmid described above along with miR- 22 mimics, inhibitors, or controls (final concentration, 50 nM). After 36 h, luciferase activity was measured using the dual-luciferase reporter assay system (Promega). For the interferon (IFN)-β, NF-κB, and IRF3 luciferase reporter assays, U251 cells were co-transfected with 100 ng IFN-β, NF-κB or IRF3 luciferase reporter plasmids together with 10 ng of the internal control plasmids, miR-22 mimics, inhibitors, or controls (final concentration, 50 nM) for 24 h, and then transfected with poly(I:C). The data are expressed as relative *Renilla* luciferase activity normalized to the value of firefly luciferase, and are representative of three independent experiments.

### RNA extraction and quantitative real-time PCR

Total RNA in treated cells was extracted with TRIzol (Invitrogen), and 1 μg RNA was used to synthesize cDNA using a first-strand cDNA synthesis kit (TOYOBO). Quantitative real-time PCR was performed using a 7500 Real-time PCR System (Applied Biosystems) and SYBR Green PCR Master Mix (TOYOBO). Data were normalized to the level of β-actin expression in each sample. To quantify mature miRNA expression, a commercial Bulge-Loop miRNA quantitative reverse transcription (RT)-PCR detection method was used. Briefly, 1 μg total RNA was used as the template and reverse transcribed using a miR-22–specific RT primer. The resulting cDNA was used for quantitative real-time PCR with a universal reverse primer and a specific forward primer. Amplification was performed for 2 min at 50°C and 10 min at 95°C, followed by 40 cycles of 95°C for 15 s, 60°C for 15 s, and 72°C for 30 s. The relative expression of miRNAs was normalized to that of internal control U6 small nuclear RNA within each sample using the 2^−ΔΔCt^ method. Expression was then standardized to the miRNA levels in mock or control miRNA–treated cells. Primers used are listed in Table 2.

### Immunoblotting

Total cellular lysates were prepared using radioimmunoprecipitation assay buffer (Sigma) containing protease and phosphatase inhibitors (Roche). Radioimmunoprecipitation assay buffer contains 150 mM NaCl, 1.0% IGEPAL™ CA-630, 0.5% sodium deoxycholate, 0.1% SDS, and 50 mM Tris with pH 8.0. For preparation of total cell extracts, PBS-washed cells were treated with ice-cold radioimmunoprecipitation assay buffer for 20 min, and were subjected to vortex after every 5 min in an ice water bath. Later, the samples were centrifuged at 12,000 *g* for 20 min at 4°C. The cleared supernatant was collected and used as the whole cellular lysate. Cytosolic and nuclear extracts were prepared using NE-PEP Nuclear and Cytoplasmic Extraction Reagent (Thermo Scientific). Protein concentrations were measured using the BCA Protein Assay kit (Thermo Scientific). Samples were subjected to SDS-PAGE, and protein bands were transferred to a polyvinylidene fluoride membrane (Millipore) using a Mini Trans-Blot Cell (Bio-Rad). Blots were probed with relevant antibodies, and positive signals were detected using ECL reagents (Thermo Scientific).

### Plaque assay

U251 cells were transfected with miR-22 mimics, inhibitors or their controls (final concentration, 50 nM) for 24 h, and subsequently infected with JEV at MOI of 5. At 12, 24, and 36 h post-infection, cell supernatants were harvested, serially diluted, and then used to inoculate monolayers of U251 cells. After removal of unbound JEV virus particles, U251 cells were further incubated for 3 to 5 days, and plaques identified. The visible plaques were counted and viral titers calculated. All data are expressed as the mean of triplicate samples.

### ELISA

The culture supernatants were collected from the treated cells at the indicated time points and stored at −80°C. The protein levels of TNF-α, IL6, and IL-1β in cell cultures were determined by ELISA kits (eBioscience) following the manufacturer's instructions.

### Statistical analysis

All experiments were carried out at least three times with similar results. Analyses were conducted using Prism 5 (GraphPad Software, San Diego, CA). Results are expressed as the mean ± SD. Data were compared with two-way analysis of variance with subsequent t tests using the Bonferroni pos*t-test* for multiple comparisons, or with the Student's *t* test. For all tests, *p* < 0.05 was considered significant.

## SUPPLEMENTARY MATERIALS FIGURE


