# Supplementary material for: MicroRNA-22 negatively regulates poly(I:C)-triggered type I interferon and inflammatory cytokine production via targeting mitochondrial antiviral signaling protein (MAVS)
Source: Oncotarget. 2016 Oct 1;7(47):76667–83. doi: 10.18632/oncotarget.12395 (PMC5363539; doi:10.18632/oncotarget.12395)
Supplement: Supplementary file 1 [file oncotarget-07-76667-s001.pdf]

MicroRNA-22 negatively regulates poly(I:C)-triggered type I interferon and inflammatory cytokine production via targeting mitochondrial antiviral signaling protein (MAVS)

Supplementary Materials

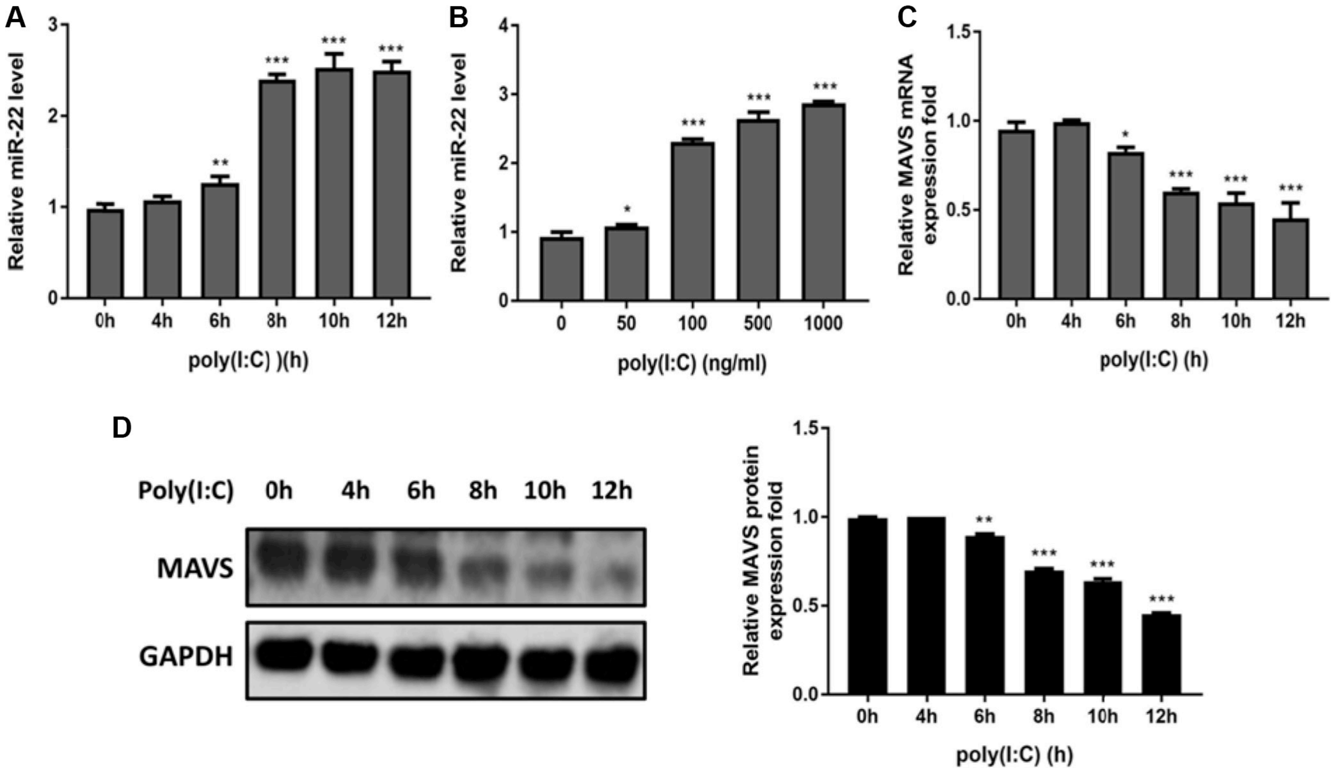

Supplementary Figure S1: miR-22 is upregulated in poly(I:C)-treated SH-SY5Y cells. (**A** and **B**) Human SH-SY5Y cells were transfected with 100 ng/ml poly(I:C) for different periods (A) or with different concentrations for 6 h (B), and miR-22 level was determined with quantitative real-time PCR. (**C** and **D**) SH-SY5Y cells were transfected with 100 ng/ml poly(I:C) for different periods, and MAVS mRNA (C) and protein (D.) levels were determined with quantitative real-time PCR and immunoblotting, respectively. Data represent means ± SD from three independent experiments. *$p < 0.05$; **$p < 0.01$; ***$p < 0.001$. Protein levels were quantified with immunoblot scanning and normalized to the amount of GAPDH expression.
